# What Pediatric Dentists Need to Know about Coronavirus Disease (COVID-19)

**DOI:** 10.30476/DENTJODS.2020.87278.1249

**Published:** 2020-12

**Authors:** Kanupriya Rathore

**Affiliations:** 1 Dept. of Pedodontics, All India Institute of Dental Sciences, Jodhpur, India

**Keywords:** Children, Coronavirus, COVID-19, Dentistry, Pediatric dentistry

## Abstract

COVID-19, a viral disease fatal yet preventable is caused by a newly identified β-corona virus. The people most vulnerable to this infection are the ones with a prior history of diseases, low immunity, or either too old or too young (particularly children and infants). In the context of the virus’s impact on the pediatric age groups, this article highlights some of the challenges and guidelines on managing it. Pediatric groups, like everyone else, are highly vulnerable to the infection by COVID-19. The lower number of pediatric patients involved at the beginning of a pandemic does not necessarily mean that children are less vulnerable to the infection. However, the good news is that the disease usually has a mild course and appropriate prevention and oral health management in children can help to keep it at bay. Adherence to simple compliance and safety protocols can go a long way. For instance, in the course of some of the procedures performed by a pediatric dentist, there may be a risk of aerosols being generated, which in turn can lead to cross-infection making the patient vulnerable to contracting COVID-19. In such a situation, parents are advised to take good home-based care and take telemedicine consultation immediately. This article lays a concrete emphasis on reviewing the limited published literature that is specific to the pediatric population regarding epidemiology, pathogenesis, diagnosis, and treatment modalities of COVID-19. It analyzes the potential risk from COVID-19 associated with pediatric dental treatment. In addition, it presents a series of considerations on potential oral prevention strategies on the management of urgent and non-urgent dental procedures in a context of disease transference control. This literature review also gives an insight for the private practitioner to have healthier management in the pediatric fraternity during this highly contagious COVID-19 pandemic

## Introduction

Severe acute respiratory syndrome coronavirus 2 (SARS-CoV-2), which causes coronavirus disease (COVID-19) emerged from Wuhan, Hubei province, China, in late 2019 and has now reached pandemic status [ 1 ]. Children at all age groups appeared susceptible to COVID-19, and there was no significant gender difference. Although clinical manifestations of COVID-19 in children patients were generally less severe than that in adult patients, young children, particularly infants, were vulnerable to infection [ 2 ]. 

Although studies have reported the standard operating protocol to manage the adult patients during the COVID-19 pandemic, the available literature does not elucidate the guidelines to be followed by the pediatric dentists while managing the pediatric population. Hence, this prompted us to analyze the available literature, compile the same for the pediatric population, and suggest recommendations and precautions for pediatric dentists during this pandemic.

### Epidemiology 

The world is currently dealing with a global outbreak of COVID-19, which is the worst humanitarian crisis we have faced since World War II. On March 11, the World Health Organization (WHO) advised that this disease presented the characteristics of a pandemic [ 3 ]. WHO defines a pandemic as the worldwide spread of a new disease [ 4 ]. Recent WHO data indicates that there are approximately 15.8 million confirmed cases of COVID-19, including 0.6 million deaths worldwide [ 5 ]. In India alone, there have been an estimated 1.4 million cases until date [ 5 ]. Although the incidence is increasing every day, compared to adults, there have been a significantly smaller number of reported cases of COVID-19 in the pediatric population. Among the total number of positive cases, in China, only about 2.4% occurred in those under 19 years of age [ 6 ]. The first pediatric case was reported on January 20, 2020, in a 10-year-old boy from Shenzhen, China, whose family had visited Wuhan City [ 7 ]. Limited data are available on the prevalence of COVID-19 in pediatric populations because children were rarely tested for the virus in the earlier phase of the outbreak, especially in Hubei Province in China, where the most cases were confirmed [ 8 ]. The youngest affected patient was a 36-hour-old newborn, who remains the youngest COVID-19 patient worldwide [ 9 ]. 

### Pathogenesis

Infection is acquired by either inhalation of droplets or touching surfaces contaminated and then touching the nose, mouth, and eyes. While data on the incubation period for COVID-19 in the pediatric population is limited, it is thought to extend to 14 days, similar to adult patients with COVID-19 [ 10 ]. In studies from China, the reported incubation period among pediatric patients ranged from 2 to 10 days [ 11 - 12 ]. The first step in infection is virus binding to a host cell through its target receptor. Human angiotensin-converting enzyme 2 (ACE2) is a functional target receptor used by 2019-nCoV to invade cells, which may promote human-to-human transmission [ 13 ]. This process requires priming by cellular serine proteases (TMPRSS2), which allow spike protein cleavage, regulating the entire mechanism [ 14 ].

The organs with high ACE2-expressing cells should be considered a potential high risk for 2019-nCoV infection [ 15 ]. ACE2 cells are abundantly present in type II alveolar cells (AT2) of lung [ 15 ], esophagus upper and stratified epithelial cells, absorptive enterocytes from the ileum, and colon [ 16 ], cholangiocytes [ 17 ], myocardial cells, kidney proximal tubule cells, and bladder urothelial ells [ 15 ]. Although COVID-19 infection hardly presented oral symptoms, the ACE2 expression in the oral cavity indicated that the oral infection route of SARS-CoV-2 could not be excluded. The presence of the ACE2 receptor of COVID-19 on the epithelial cells of the oral mucosa, especially in epithelial cells of the tongue, might provide possible routes of entry for the SARS-CoV-2, which indicates oral cavity might be a potential risk route of SARS-CoV-2 infection [ 18 ]. The results of a study conducted in 2020 by Xu *et al*. [ 19 ] revealed a huge expression of the ACE2 receptor of COVID-19 on the epithelial cells of the oral mucosa, especially in epithelial cells of the tongue. ACE2 epithelial cells of salivary gland ducts were demonstrated to be early targets of SARS-CoV-2 infection [ 19 ]. 

### Possible transmission routes of 2019-nCov in dental clinics

The common transmission routes of novel coronavirus include direct transmission (cough, sneeze, and droplet inhalation transmission), contact transmission (contact with oral, nasal, and eye mucous membranes), and airborne spread (droplet and aerosol transmission) [ 20 ]. 

### Contact spread

Dental care settings invariably carry the risk of SARS-CoV-2 infection due to the specificity of its procedures, which involves face-to-face communication with patients. The infection can be transmitted in dental settings through inhalation of airborne microorganisms that remain suspended in the air for long periods. A dental professional’s frequent exposure to blood, oral fluids, or other patient materials [ 21 ], makes a possible route for the spread of viruses. In addition, dental professionals are at a risk of being exposed to conjunctival, nasal, oral mucosa through droplets and aerosols from an infected individual [ 22 ]. 

### Contaminated surfaces spread

Human corona viruses can remain infectious on inanimate surfaces like metal, glass, or plastic at room temperature for up to 9 days [ 23 ]. Frequent contact with these inert surfaces in healthcare settings is therefore a potential source of viral transmission [ 23 ]. 

### Spread via Dental procedures

Dental procedures inadvertently generate aerosols. Dental procedures that use low- or high-speed handpieces, lasers, electrosurgery units, ultrasonic scalers, air polishers, prophy angles, hand instruments, or air/water syringes can create bioaerosols and spatter. Ultrasonic scalers and high-speed handpieces produce more air borne contamination comparatively [ 24 ]. 

### Diagnosis

The diagnostics can play a pivotal in restraining and spread of COVID-19 [ 25 ]. A proper contact history, systemic symptoms, and radiographic changes of pneumonia can help in establishing the provisional diagnosis but for the final diagnosis laboratory investigations are more reliable [ 26 ]. For evaluating or diagnosis of early infection, the WHO suggests the rapid collection and nucleic acid amplification testing (NAAT) of respiratory samples including nasopharyngeal swabs and oropharyngeal swabs as well as sputum specimen and/or endotracheal aspirate or bronchoalveolar lavage [ 27 ]. Laboratory tests, chest X-ray scan, chest computed tomography, lung ultrasound findings are rather essential in early screening of suspected cases and diagnosis of COVID-19 [ 28 ].

According to the WHO, the immediate priority for COVID-19 diagnostics research is the development of nucleic acid and protein tests and use of point-of-care immunodiagnostic tests [ 6 ]. These simple test kits are based on either detection of proteins from the COVID-19 virus in respiratory samples (e.g. sputum, throat swab) or detection, in blood or serum, of human antibodies generated in response to infection. Based on current evidence, WHO recommends the use of these new point-of-care immunodiagnostic tests only in research settings [ 29 ]. Point-of-care tests are cost-effective, hand-held devices used to diagnose patients without sending samples to centralized facilities. These can be feasible in community centers to reduce the burden on clinical laboratories [ 30 ]. Lateral flow antigen detection [ 31 ] and micro fluidics-based smart phone [ 32 ] are the tests under development for diagnosing COVID-19. Other diagnostic technologies that have shown clinical feasibility are discussed in a review article by Udugama *et al*. [ 33 ].

### Management of COVID-19

Development of therapeutics and vaccines is underway and currently there is no approved therapeutics or vaccines approved by the U.S. Food and Drug Administration (FDA) to prevent or manage COVID-19 infection [ 25 ]. Ongoing clinical management includes infection prevention and supportive care, including supplemental oxygen and mechanical ventilator support when indicated. The only FDA-recommended drug is “remdesivir”. Well-studied scientific tests are required to appraise the effectiveness and safety of other antiviral agents, chloroquine, and hydroxychloroquine, corticosteroids, convalescent plasma therapy, and vaccines in patients with COVID-19 infection [ 26 ]. There are over 100 projects trusted sources around the world centered on the development of a COVID-19 vaccine [ 34 ] Vaccine development is a long process, and currently no COVID‐19 vaccine has been successfully developed. Trivedi *et al*. [ 35 ] in their review article, have summarized the medicines currently being investigated in clinical trials for the potential treatment of COVID-19 and has highlighted the potential benefits and issues of their use in clinical practice. Although the eventual results of these studies will take a long time to accomplish, the provisional research data may provide some help for the present urgent demand for therapy [ 28 ].

## Literature Research

### Databases

The electronic search was undertaken by searching the PubMed and Advanced Search (Basic Search) catalog to search evidence-based clinical practice guidelines. The databases have been searched from January 1 up to July 27 2020 using the following search terms: “COVID” and “children”, “COVID” and “Dentistry”, “COVID” and “Pediatric dentist”, “COVID” and “dental infection control”, “COVID” AND “dental guidelines”, “COVID” and “recommendation for a dentist”, “COVID” and “children” and “Dentistry”. A Google search has also been carried out to identify any recommendations for dental practice and dental guidelines.

### Inclusion and exclusion criteria

We included all studies that have reported the recommendation and practice guidelines for general and pediatric dentists during COVID-19. The electronic search was complemented with a hand search of the following websites: American Dental Association, American Academy of Pediatric Dentistry, Alberta dental association and college, European Academy of Paediatric Dentistry, British Society of Paediatric Dentistry, Australian Dental Association, Indian Dental Association, Ministry of health and family welfare, Iowa Dental Association, International Association of Paediatric Dentistry, Scottish Dental Clinical Effectiveness Program, Royal College of Surgeons, Centers for Disease Control and Prevention, and Google Scholar. The excluded articles were published in languages other than English, double publications, abstracts only, and textbooks.

## Discussion

### Is the pediatric population less susceptible to COVID-19?

The face of epidemics and pandemics has changed over the years. Humankind has overcome these pandemics in the past and the evolving immune system has helped them survive. Since SARS-CoV-2 is a new zoonotic pathogen, there is no pre-existing immunity and the whole of humanity is susceptible to infection and developing COVID-19 disease [ 36 ]. 

Children can also be infected by SARS-CoV-2, but most pediatric cases with laboratory-confirmed SARS-CoV-2 infection are mild; severe COVID-19 disease in children is rare [ 36 ]. The immune preparedness of children to any novel pathogens, including, SARS-CoV-2 might be based on several factors, as described below:

- Children have a more active innate immune response.
- The exposure to cigarette smoke and air pollution is less in children as compared to adults. Thus, they have healthier respiratory tracts and fewer underlying disorders [ 37 ]. 
- Another suggested explanation is that viral interference in the respiratory tract of young children leads to a lower viral load in children. 
- The ACE2 receptor for the SARS-CoV-2 virus may be expressed differently in the fetal lung compared to adult lung tissue [ 38 ]. ACE2 expression in rat lung has been found to decrease with age dramatically [ 39 ]. As reported by Hoffmann *et al*., fetal lung AC-E2 receptors have different characteristics than mature lung tissue (e.g., lower binding capacity) [ 14 ]. 
- The children are exposed to other respiratory viruses such as respiratory syncytial virus, influenza A and influenza B viruses, which enhance their serum anti-body levels and could, provide cross-protection [ 38 ]. 
- Children have fewer outdoor activities and undertake less international travel, making them less likely to contract the virus [ 40 ]. 

### Recommendations for pediatric dentists

Several precautionary steps should be taken by the dental healthcare professionals to be able to function in the “new normal” environment. While the previous transmission pathways are common to the treatment of any dental patient, pediatric patients present additional risks of transmission. The use of removable orthodontic appliances or auxiliary elements in fixed orthodontic therapies, such as the use of intermaxillary elastic bands, entails risks of contamination if handling is not carried out with due precautions. Another problem is related to the difficulty for the child to don personal protective equipment (PPE) during medical visits. Finally, the very presence of caregivers, with whom the pediatric dentist must unavoidably interface, will increase the risk of infection [ 41 ]. 

According to a study [ 42 ] conducted by Huaqiu Guo *et al*., the proportion of dental and oral infections increased from 51.0% before the COVID-19 outbreak to 71.9% during COVID-19. Thus, there is evidence to believe that in the post-COVID-19 era, people's demands for dental services may extremely rise. Hence, as frontline health care professional, dentists have to be more careful and the dental operatories should gear themselves for readiness [ 42 ]. 

### General recommendations for management of pediatric patient 

Evolution has endowed a survival advantage to children to combat known and unknown pathogens; children can be asymptomatic or present with mild, nonspecific symptoms. Because of the long incubation period, ranging from 2-14 days (mean 6 days), all child patients, and parents should be considered potential carriers of COVID-19 unless proved otherwise [ 43 ]. Taking into account findings of previous literature [ 44 - 46 ], a set of general suggestions listed below are aimed at safety as well as operational changes consistent with what is known and what is still emerging about the COVID-19 pandemic.

- Child should go into the dental operatory alone and their parent should be asked to wait outside to reduce the number of individuals in the clinical area.
- Always use the appropriate type of PPE. 
- There should be a dedicated area for donning and doffing of PPE.
- Implement four-handed dentistry techniques
- Prioritize the use of hand instrumentation, to reduce aerosol production as much as possible
- Use a high volume aspirator to minimize aerosol generation.
- Isolate the operating field with the rubber dam to minimize the production of aerosols contaminated with blood and saliva, especially during the treatment of the pulpits.
- Use high-speed turbines with an anti-retraction valve to reduce the return flow of oral bacteria.
- Minimize the use of a 3-in-1 syringe as this may create droplets due to forcible ejection of water/air.
- Use of extraoral dental radiographs during the COVID-19 should be encouraged in order to enhance patient experience, as intraoral dental radiographs can stimulate aerosol generation from saliva secretion, gagging, coughing, or vomiting especially in children [ 47 ].
- To avoid a follow-up appointment, use resorbable sutures.
- After any type of treatment, properly clean surface areas and reusable equipment, as well as clean up and dispose of hazardous waste.
- Maintain air circulation with natural air through a frequent opening of windows and employ an independent exhaust blower to extract the room air into the atmosphere.
- Indoor portable air cleaning system equipped with HEPA filter and UV light may be used.
- Digital payment (paperless/contactless) methods are preferred.
- Promoting the use of social digital technology platforms (e.g. social media-based app, YouTube etc) for educating the parents and children for oral health promotion would be effective in improving oral health and oral hygiene.
- Diet counseling should be done for inculcating correct dietary habits, and home oral hygiene measures like regular use of mouthwash and twice brushing should be promoted to avoid new dental cavities.
- Remove hard-to-disinfect items from operatories and waiting area (toys, decorations, books, magazines).

### Tele-consult and Tele-screening Triage

Primary telephone screening to recognize suspected patients or probable COVID-19 infection can be done before scheduling appointments. Questions related to any travel history to COVID-19 infected regions, the existence of febrile respiratory illness (FRI) symptoms such as cough and fever, or presence of other concomitant diseases should be asked during telephone screening. A positive answer to any of these two questions would increase the initial concern and postpone the elective dental care for at least two weeks [ 48 ]. 

Dental clinics should establish a pre-check triage to make a recording of the temperature of both the child and their caregivers. When the patient visits the clinic, a retrospective evaluation should be carried out to investigate not only trips to geographic areas affected by the pandemic of COVID-19 made in the 14 days but also if there is a history of contact with COVID-19 confirmed or suspected patients. The child should be accompanied by a minimum number of people. In addition to measuring the temperature, medical protective masks should be provided to patients and their caregivers before entering the operatory [ 49 ].  

### Instruction to be followed by patients before arrival at a dental clinic [ 49 - 52 ]

- Avoid wearing a wristwatch, hand, body jewelry, and carrying of additional accessories bags and so on. 
- Use their washrooms at home to avoid the need of using toilets at the dental facility. 
- Pre-procedural use of mouthwash should be advocated. Oxidative agents containing mouth rinses with 1% hydrogen peroxide or 0.2% povidone-iodine are recommended [ 52 ]. 
- Wear a facemask during transport and before entering the premises.
- Have the body temperature checked and use a sanitizer on the entrance. 
- Patients consent and declaration to be obtained in a physical print out or electronic system. 
- If the waiting room does not allow for appropriate “social distancing” (situated at least 6 feet or 2 meters apart), patient may wait in their vehicle or outside the facility where they can be contacted by a mobile phone when it is their turn to be seen [ 49 ]. 
- Respiratory hygiene and cough etiquette should be practiced [ 51 ]. 

### Patient follow-up and Review 

The patient should be followed up after the dental treatment. Patients should be contacted by telephone 24hrs and in a week to find out if they have developed any symptoms that should warn the dental staff to undertake appropriate actions. Patients should be advised to inform back to the dental clinic if they develop anyadverse symptoms [ 50 ]. 

### Specific recommendation for management of different dental problems 

Pediatric dental practice challenges us to prepare for situations that may not occur for general dental practices or other specialties. For the management of different pediatric patient groups refer to the practice checklist by the American Academy of Pediatric Dentistry [ 46 ]. 

### Management of dental pathologies that do not represent an emergency

No routine dentistry should be provided for children during this pandemic. In agreement with the previous literature [ 41 ], ADA suggested in its Interim Guidance [ 45 ], to delay the treatment of non-urgent dental proc-edures that do not require emergency treatment indefinitely during the COVID-19 acute phase; following pr-ocedures are enumerated below (Table 1 and Table 2).

**Table 1:** Recommendation for Dental care during COVID-19 Pandemic for different dental specialties [53-57]

Dental treatment	Dental emergency	Urgent Care	Dental non emergency
Pedodontics	• Children with underlying medical conditions, which place them at greater risk of complications arising from any subsequent infection if the tooth is not treated require emergency treatment	• Presence of a swelling likely to or compromising swallowing and/or breathing, causing trismus or extending to the eye or a significant oral/facial swelling with associated pyrexia.	• Deciduous / permanent teeth affected by previous carious lesions and treated with temporary dressings:
	• Children and young people with additional needs such as those with learning disabilities or autism, where dental pain is resulting in self-harm or other disruptive or detrimental behaviors.	• Traumatic dental injuries resulting in a complex injury to the permanent dentition: avulsion of a permanent tooth; severe luxation, crown root fracture, complicated crown fracture. Traumatic dental injuries to the primary dentition resulting in: pulp exposure or severe luxation such that tooth mobility constitutes a potential airway risk and/or is severely interfering with occlusion/function.	• Delays of deciduous teeth exfoliation with their persistence in the arch, in conjunction with the simultaneous eruption of the corresponding permanent tooth
	• Increased risk of infection (e.g., any immunocompromised state, transplant patient, diabetic, and child on immunosuppressant /steroids/chemotherapy).		• Eruptive gingivitis of the permanent first molar
	• Children whose poor dental health is impacting on, or is highly likely to impact on, their medical health		• Malocclusions associated with crowding of the dental elements and with overjet and overbite alterations
	• Increased risk of bleeding from medications or conditions.		
Endodontics	• Active infection with pus and swelling associated with pain which cannot be managed by over the counter medications	• Severe dental pain from pulpal inflammation	• Non-painful chronic periapical lesions
	• Swelling or cellulitis – only access opening and medication has to be administered and appointment needs to be postponed	• Abscess, or localized bacterial infection resulting in localized pain and swelling.	
	• Change of interim restoration in case of severe pain in patients with access opening.	• Replacing temporary filling on endo access openings in patients experiencing pain or an endodontically treated tooth with a high fracture potential	
Oral surgery	• Uncontrolled bleeding	• Pericoronitis or third-molar pain	• Postpone asymptomatic third molar surgeries
	• Cellulitis or a diffuse soft tissue bacterial infection with intra-oral or extra-oral swelling that potentially compromises the patient’s airway.	• Surgical post-operative osteitis	• Extraction of asymptomatic teeth
	• Trauma involving facial bones, potentially compromising the patient’s airway oral-facial trauma.	• Dry socket dressing changes	
		• Dental trauma with avulsion/luxation	
		• Tooth fracture resulting in pain or causing soft tissue trauma	
		• Suture removal	
		• Biopsy of a suspicious oral lesion or abnormal oral tissue	
Orthodontics		• Adjustment of orthodontic prosthesis	• New patients for bonding, recall, consultations.
		• Managing active orthodontic cases	
		• Snipping or adjustment of orthodontic wire/appliances ulcerating the oral mucosa.	
Restorative		Extensive dental caries or defective restorations causing pain	Treatment of asymptomatic carious lesions
Periodontics			Routine dental cleaning and preventive therapies
Prosthodontics		Final crown/bridge cementation if the temporary restoration is lost, broken or causing gingival irritation	Postpone replacement of crowns for decayed tooth or missing teeth
		Denture adjustment on radiation/oncology patients	
		Denture adjustments or repairs when function impeded	
Others	Management of known/high risk malignancy.	Dental treatment required prior to critical medical procedures	Dental implants
	Active sleep apnea management		Initial or periodic oral examinations and recall visits, including routine radiographs, cosmetic/ aesthetic (Bleaching, laminates and veneers)
	Pre-surgical clearance for medical procedures		

**Table 2:** Management of Acute Dental Problems During COVID-19 Pandemic [53-57]

Problem (symptoms)	Ways of Management		
	Advice & Self Help	Urgent Care	Emergency Care
• Acute apical abscess	• Recommend optimal analgesia.	If patient has spreading infection without airway compromise, or patient has continuing or recurrent symptoms, refer to urgent dental care centre for extraction/ drainage.	If patient has spreading infection with or likely to have airway compromise and/or severe trismus
• Acute periodontal abscess/Perio-endo lesions	• Prescribe antibiotics if you are concerned about swelling or if there are signs of systemic infection (fever, malaise)		
• Acute pericoronitis	• Ask patient to call back in 48-72 hours if their symptoms have not resolved.		
Necrotizing ulcerative gingivitis/periodontitis	• Recommend optimal analgesia.		
	• Recommend chlorhexidine or hydrogen peroxide mouthwash.		
	• Give oral hygiene advice (benzydamine mouthwash or spray may make tooth brushing less painful).		
	• Consider antibiotics (metronidazole is drug of first choice)		
Reversible pulpitis	• Recommend optimal analgesia.		
	• If due to a missing filling, advise patient to use an emergency temporary repair kit, which can be purchased online or at a pharmacy.		
	• Advise patient to avoid hot and cold food.		
	• Advise patient to call back if symptoms get worse		
Irreversible pulpitis	• Recommend optimal analgesia.	If pain is severe and uncontrollable, preventing sleeping or eating, refer to dental care centre for management/extraction.	
	• Advice patient to try rinsing with cold water as this can alleviate pain.		
	• Advise patient to call back if symptoms get worse.		
Oral ulceration	• If ulceration has been present for less than 3 weeks: advise chlorhexidine mouthwash (not for <7 years of age);	If ulceration has been present for 3 weeks or more, refer the patient to designated urgent dental care centre.	If a patient with oral ulceration is severely dehydrated, refer for emergency medical care.
	• Recommend optimal analgesia including topical analgesics (e.g. benzydamine oro mucosal spray)		
	• Recommend soft diet;		
	• If due to trauma from adjacent tooth, advise patient to use an emergency temporary repair kit.		
	• In cases of primary herpetic gingivostomatitis or herpes zoster infection, if the symptoms are severe or the patient is immunocompromised, consider prescribing antiviral agents (acyclovir or penciclovir), ideally in the early stages.		

### Management of acute dental problems requiring urgent and emergency care 

According to Scottish Dental Clinical Effectiveness Programme guidelines [ 53 ] for “Emergency Dental Care and Management of Acute Dental Problems” management can be provided in three ways:

- Advice and self-help: mild and moderate symptoms managed remotely by general dental practices providing advice and self-help, which might involve analgesics and antimicrobials
- Urgent care: severe or uncontrolled symptoms that cannot be managed by the patient and require the patient to see a dentist in a designated urgent dental care center
- Emergency care: emergency conditions that require immediate medical attention

Based on the guidelines highlighted in the literature [ 53 - 57 ], the following key points are tabulated below (Table 1 and Table 2).

### Management of orofacial trauma

Traumatic pathology has a prevalence that varies between 6.1% and 62.1% in individuals of preschool age [ 58 ] and between 5.3% and 21% of school-age [ 59 ]. It is therefore likely that it can present itself as an emergency to be managed during the COVID-19 pandemic. In general, the planning of the treatment of dental fractures, dislocation, or dental avulsion depends on the age, the traumatic severity of the dental tissue, the development of the apex, and the duration of the dental avulsion [ 60 ]. The management protocols for orofacial trauma have been tabulated below (Table 3) [ 53 ]. 

**Table 3:** Recommendation for Management of Traumatic dental injuries during COVID-19 Pandemic [53]

Problem (symptoms)	Ways of Management		
	Advice & Self Help	Urgent Care	Emergency Care
Dento-alveolar injuries	• If the patient is not in need of emergency medical attention, advise them to:		• If bleeding is severe and will not stop within 15-30 minutes
	• Clean the affected area by rinsing gently with mild antiseptic and if foreign object(s) are present in the mouth, remove them		• There has been significant facial trauma
	• Apply ice packs to soft tissue injury and swelling; apply pressure with a finger to stop any bleeding		• Patient has had a head injury or loss of consciousness
	• Consider recommending analgesia		• Patient has inhaled a tooth or tooth fragment.
	• Do not prescribe antibiotics		
Avulsed, displaced or fractured teeth	• If a permanent tooth fracture involves only enamel and dentine, advise the patient to apply desensitizing toothpaste on the exposed dentine and to use an emergency temporary repair kit, which can be purchased online or at a pharmacy.	• If a permanent tooth has been knocked out, advise the patient to	• If bleeding is severe and will not stop within 15-30 minutes
	• If a primary tooth (or teeth) has been knocked out or displaced without affecting the bite, advice the parent/caregiver to alter the child’s diet to include soft food and appropriate analgesia if required.	• Handle the tooth by its crown and avoid touching the root;	• There has been significant facial trauma
	• Note. Primary teeth should not be re-implanted.	• If the tooth is dirty, wash it briefly (10 seconds) under cold running water	• Patient has had a head injury or loss of consciousness
		• Try to re-implant the tooth in its socket and then bite gently on a handkerchief to hold it in position;	• Patient has inhaled a tooth or tooth fragment.
		• If this is not feasible, store the tooth for transportation in milk (not water). Alternatively transport the tooth in the mouth, keeping it between molars and inside of the cheek.	
		• Urgent management is required if a permanent or primary tooth is out of occlusion and is affecting the bite,	
		• If a tooth fractures involving the pulp	

### Considerations for treating patients with suspected or confirmed COVID-19 [ 61 ]

- Dental treatment of COVID-19 suspected or confirmed patient should be done in a room with a closed door.
- Avoid aerosol-generating procedures if possible.
- Proper precautions should be taken while performing aerosol-generating procedures, which are already discussed under “General recommendations for management of pediatric patient”.
- COVID-19 positive patient should be scheduled at the end of the day and no other patient should be appointed at that time.
- Management of hospitalized COVID-19 positive children with underlying medical conditions and those who are immunocompromised should be done on urgent basis as discussed in Table 3.
- In case of severe dental emergencies, for uncooperative children, dental treatment should be performed in public hospitals under sedation and/or general anesthesia to provide a safe and effective dental treatment [ 62 ].

## Conclusion

Dentists who treat children amidst the coronavirus pandemic should assume that every person is potentially infected and they should indisputably follow universal infection control procedures. It is essential that in the present scenario, priority be given to dental procedures labeled as emergencies by the WHO. This would be an appropriate step in attempt to curtail the further spread of COVID-19. This is also a huge opportunity to motivate children to maintain good oral health and promote preventive dental behaviors in them. Parents should be educated regarding the home management of milder oral pathologies for which direct intervention of the specialist is not necessary or can be deferred until a time when the outbreak goes into recession. The specialists in pediatric dentistry should promote the use of tele-consultation and conte- mporary minimally invasive procedures that minimize or eliminate aerosol generation during this pandemic.

While we all work towards curbing this outbreak, efforts should be made to devise comprehensive2 measures to prevent future outbreaks of zoonotic origin. It is hoped that the compiled guidelines discussed in this article will help in the management of pediatric dental patients globally during this COVID-19 pandemic, and provide a solid base for further healthcare guidelines development.
